# Isolated extrahepatic bile duct rupture: a rare consequence of blunt abdominal trauma. Case report and review of the literature

**DOI:** 10.1186/1749-7922-7-16

**Published:** 2012-05-24

**Authors:** Ruben Balzarotti, Stefania Cimbanassi, Osvaldo Chiara, Gianpietro Zabbialini, Claude Smadja

**Affiliations:** 1 Department of Emergency Surgery and Trauma Center, Niguarda Ca’ Granda Hospital, Piazza Ospedale Maggiore 3, 20162, Milan, Italy; 2 Department of Surgical Oncology, Treviglio-Caravaggio Hospital, P.le Ospedale 1, 24047, Treviglio, BG, Italy; 3 Department of Digestive Surgery, Antoine Béclère Hospital, 157 rue de la Porte de Trivaux, 92141, Clamart Cedex, France

**Keywords:** Blunt abdominal trauma, Hepatic injury, Extrahepatic bile duct rupture

## Abstract

A 16-year-old girl suffered blunt abdominal trauma. Clinically, a severe motor impairment with paraesthesia of the legs was found. Posterior osteosynthesis in T10-L1 with laminectomy in T10-T12 and posterolateral arthrodesis in T11-T12 was performed because of a dorsal traumatic vertebral fracture. On hospital day 7, because of an acute abdomen, surgical laparoscopic exploration showed sterile bloody fluid without any evident hemorrhagic injury. On hospital day 11, the patient was reoperated on by the laparoscopic approach for increasing abdominal pain and fever: a peritoneal biliary fluid was aspirated. After conversion to open surgery, cholecystectomy was performed. Intraoperative cholangiography was considered as normal. On arrival at our institution 13 days after injury, the patient was operated on for a biliary peritonitis. Intraoperatively, a trans-cystic cholangiography showed a biliary leakage of the common bile duct; a T-tube was placed into the common bile duct; a subhepatic drainage was placed too. On postoperative day 30, a T-tube cholangiography showed a normal biliary tree, without any leakage, and the T-tube was subsequently removed. The patient had a complete recovery.

## Background

Common bile duct (CBD) injuries from blunt abdominal trauma are rare [1]. In fact, extrahepatic biliary tract injuries occur in 3% to 5% of all abdominal trauma victims, with 85% resulting from penetrating wounds. Of the remaining 15%, resulting from blunt trauma, the vast majority, 85%, involve the gallbladder alone.

Injury of the extrahepatic biliary system after blunt trauma is a relatively rare entity. The first report of bile duct rupture was in 1799 by Wainwright [2,3]. Bourque et al [4] in his review of the literature in 1989 found only 125 cases reported since 1806, one third of which were in the pediatric population. Dawson et al [5] reported 1 case of bile duct injury in 10,500 consecutive trauma patients.

Complete CBD transection is particularly rare too [6].

We report a case of an isolated extrahepatic bile duct rupture, without any associated intra-abdominal injury. It is extremely rare, and, when it occurs, concerns mainly the CBD [7]. A summary of these cases (clearly and well-documented cases without other significant associated intra-abdominal injuries, found in the English Literature), including patient age, mechanism, location of ductal injury, is supplied in Table 1.

**Table 1:** Patients with isolated extrahepatic bile duct rupture due to blunt abdominal trauma: 34 cases

**Author**	**Patient age (yr)**	**Mechanism of injury**	**Location of ductal injury**
Nikishin [8]	3	Run over by auto	RHD
Plewes [9]	6	Run over by auto	CBD
Turney [10]	39	Steering wheel	CBD
Review: 20 patients	-	-	CBD
Shorthouse [11]	8	Iron bar fell over abdomen	CBD
Janss [12]	30	Steering wheel	CBD
Rohatgi [13]	10	Fall onto handlebar	CBD
Bourque [4]	3	Sledding accident	CBD
Kim [14]	17	Fall onto handlebar	CBD
Drabble [15]	14	Motor vehicle accident	CBD
Gerndt [16]	20	Rollover motor vehicle crash	RHD
	19	Rollover motor vehicle crash	LHD
Krishnamurthy [17]	38	Assault with sticks and iron rods	CBD
Ramia [18]	36	Accidental fall in her bath	CBD
D’Amata [19]	24	-	CBD

Abbreviations: CBD, common bile duct; LHD, left hepatic duct; RHD, right hepatic duct.

## Case presentation

A 16-year-old girl suffered blunt abdominal trauma by a road traffic accident. She underwent horizontal deceleration trauma by car crash.

She was admitted to local hospital emergency room. On arrival, she had a Glasgow Coma Score of 15, and she was hemodynamically stable. An abdominal guard reaction on the left side and severe motor impairment with paraesthesia of the legs were found. Laboratory values showed hemoglobin level 11.3 g/dL, total serum bilirubin 21 μmol/l, aspartate aminotransferase 106 IU/l (normal value < 40), alanine aminotransferase 57 IU/l (normal value < 56), and prothrombin time and partial thromboplastin time of 56% and 33 seconds, respectively. An abdominal CT scan with intravenous contrast disclosed a doubtful image of traumatic splenic injury with peritoneal fluid surrounding the spleen and a dorsal vertebral fracture.

In front of a doubtful splenic injury managed non operatively, only vertebral fracture was treated: posterior osteosynthesis in T10-L1 with laminectomy in T10-T12 and posterolateral arthrodesis in T11-T12 was performed.

On hospital day 7, because of an abdomen become tense and distended with worsening discomfort, surgical exploration by laparoscopy was performed. Sterile bloody fluid (700 ml) without any evident hemorrhagic injury was found. The doubtful splenic fracture was not confirmed intraoperatively.

On hospital day 11, because of a clinical and biological deterioration with a significant increase in the hepatic cholestatic enzymes and the detection of diffuse peritoneal fluid at ultrasound, the patient was reoperated on, for the second time, by the laparoscopic approach: a biliary peritonitis was found and the peritoneal biliary fluid (1000 ml) was aspirated; some inflammatory adhesions were present in the gallbladder region. After conversion to open surgery, no evident injury was found after careful surgical exploration. Cholecystectomy with intraoperative cholangiography was performed. No evidence of bile leakage was detected.

On hospital day 13, in front of a further clinical and biological deterioration associated with a bilious fluid drained from surgical drain positioned into the subhepatic area, the patient was finally transferred to a highly specialised hepatobiliary surgical Division.

On arrival at our Institution, hemodynamic patterns of septic shock were found, associated with a bilious fluid from surgical drain and a diffuse peritoneal fluid effusion at CT scan.

**Figure 1:**
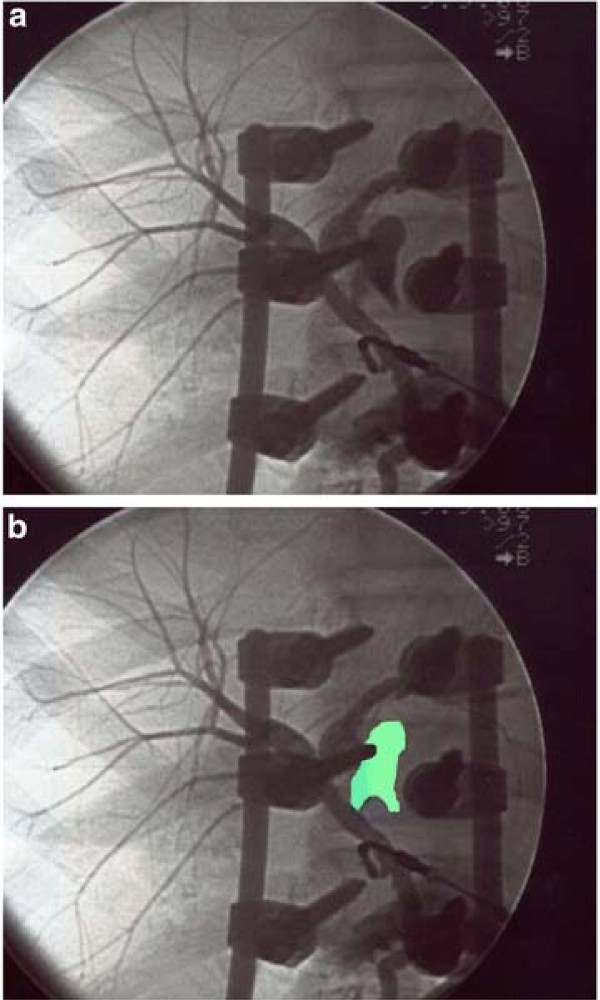
**Intraoperative trans-cystic cholangiography. a**) a biliary leakage appears on the left posterolateral aspect of the common bile duct, 1 cm below the biliary confluence; **b**) contrast material leakage is highlighted in green.

The diagnosis of post-traumatic biliary fistula with generalized peritonitis was considered, requiring urgent surgery. Intraoperatively, there was no evidence of injury; the biliary peritonitis was confirmed; peritoneal lavage was performed; a trans-cystic duct methylene blue injection was performed to look for biliary leakage, but no leak could be detected by this method; finally an intraoperative trans-cystic cholangiography, hampered by the presence of material for vertebral osteosynthesis and consequently performed in different oblique plans, allowed the detection of biliary leakage on the left posterolateral aspect of the common bile duct, one cm below the biliary confluence (Figure 1); because of inflammatory changes due to the peritonitis, the dissection and the direct approach to the biliary leak was considered hazardous; the residual cystic duct stump was excised, and a T-tube was placed into the common bile duct; a subhepatic drainage was placed too.

Over the postoperative period, the patient continued to improve steadily with gradual return of bowel function and oral feeding.

On postoperative day 30, a T-tube cholangiography showed a normal biliary tree, without neither leakage nor stricture. The T-tube was subsequently removed and the patient was discharged from the intensive care unit. The patient had a complete recovery.

## Discussion

CBD injury occurs frequently at three areas of relative fixation of the biliary tract [20]: 1) the origin of the left hepatic duct, 2) the bifurcation of the hepatic ducts, 3) the pancreaticoduodenal junction.

Different mechanisms, even in combination, may produce rupture of the common bile duct: compression of the ductal system against the vertebral column [21], sudden increase of intraluminal pressure in the gallbladder with a short and permeable cystic duct [22], and a “shearing force” producing avulsion of the common duct at its fixed part at the junction with the pancreas [23].

The diagnostic modalities to be used and the order of testing depend greatly on the stability of the patient, risk, or suspicion of associated injuries, and other indications that may necessitate operative exploration.

Diagnosis may be performed in three different moments [24]: immediately in patients undergoing laparotomy for associated injuries, lately in stable patients with scant symptoms (>50% of cases), and because of complications due to missed injuries at the time of the trauma.

Common bile duct injury is often discovered during laparotomy when bile staining in the hepatoduodenal ligament area prompts exploration. The diagnosis is often more difficult with incomplete injuries that result in a delayed presentation. These cases may present days to months postinjury, with nausea, vomiting, jaundice, and abdominal pain [25]. Such symptoms are caused by a stricture or bile leak from a direct injury or ischemic insult from injury resulting in devascularization of the extrahepatic biliary tree.

The diagnosis of a bile duct injury is often difficult in the multiply injured patient and demands a high index of suspicion. Worsening abdominal discomfort, distension, nausea, vomiting, persistent ileus, hyperbilirubinemia, and low-grade fever commonly are associated with bile duct injury, but are nonspecific. The first diagnostic test should be an abdominal ultrasound or CT scan to confirm free fluid, but a concomitant liver injury with hemoperitoneum often is present. A diagnostic peritoneal lavage with testing for bilirubin is sensitive but not specific; ERCP (contemporarily diagnostic and therapeutic, allowing positioning of a plastic stent in some settings) or magnetic resonance cholangiography defines the area of injury more precisely.

The combination of suboptimal imaging modalities, the presence of confounding injuries, and the rare incidence of blunt traumatic CBD injuries contribute to the diagnostic challenge of these problems.

Diagnostic delays have been described in patients with blunt injuries to the ductal system [26]. Those delays probably include two different conditions: real diagnostic delay because of difficulty of diagnosis and delayed onset of biliary duct trouble [27].

Late recognition and inappropriate management of these injuries result in severe, often fatal consequences [28]. Thus, any patient sustaining blunt abdominal trauma whose workup suggests possible pancreatic, liver, or duodenal injury requires a thorough evaluation.

The approach to the management of these patients depends primarily on the patient’s hemodynamic stability: unstable patients are best served with an immediate exploratory laparotomy. In the stable patient, controversy exists concerning the decision to operate based on equivocal CT findings. However, a frequent incidence of significant visceral injury has been reported with the CT finding of free abdominal fluid without evidence of solid-organ injury [29]. Patients who have persistent or worsening abdominal pain, or a persistent base deficit despite adequate resuscitative efforts, probably will often need a celiotomy.

In our case, the delay of the adequate treatment was due to the late onset or identification of an evident biliary peritoneal fluid, and to the difficulty in locating bile leakage.

In the first operation, carried out because of worsening abdominal tenderness (we could argue why any preoperative radiologic exam was not performed), only sterile bloody fluid was found. We could advocate two possible explanations: a bile leakage was already present, but not yet macroscopically evident because of the concomitant and more important hemoperitoneum of uncertain origin; differently, we can consider the late onset of the biliary leakage, some days after the hemorrhagic injury. In these two circumstances, we can image two different traumatic mechanisms: in the first case, a rapid deceleration mechanism or a direct crash, with dorsal vertebral fracture, causing a compression and the rupture of CBD, during the road accident; in the second one, a late ischemic necrosis of CBD and consequent bile leakage, due to an arterial injury responsible of hemoperitoneum. We must also consider a multifactorial mechanism, traumatic and ischemic in the same breath.

In all cases, the first laparoscopic approach was probably inadequate in order to wash and explore the abdominal cavity. The splenic rupture was not confirmed, but, suspecting that, it was probably cautious not to mobilize the spleen, neither by laparoscopic approach nor by laparotomy, in order to completely explore the spleen at all costs.

In the second operation, a peritoneal bilious fluid with peritonitis was finally detected by laparoscopic approach. Conversion to laparotomy was mandatory, in order to identify bile leak. A careful exploration of the liver and the duodenum was carried out. The presence of inflammatory adhesions in the hepatoduodenal ligament area certainly focused attention on gallbladder and CBD region. Nevertheless, no bile leakage was detected. Due to the fact that blunt abdominal trauma involve the gallbladder more often that the CBD [1], even without any sign of gallbladder rupture in the operative report, cholecystectomy was performed. This attitude can be argued. Certainly cholecystectomy was not mandatory, even for the purpose of performing a cholangiography. Probably, in presence of inflammatory changes and adhesions, first surgeon was not completely sure concerning the gallbladder integrity, and cholecystectomy was considered a safe surgical procedure, in this setting, to solve the doubt and, at the same time, to achieve intraoperative radiographic examination of the bile ducts. Cholangiography was not able to identify contrast medium leak from CBD, probably due to the presence of material for vertebral osteosynthesis. By the operative report, cholangiography was not performed in any other different view. The dissection of the porta hepatis was not attempted, probably due to the inflammatory changes and the poor surgical expertise in this field. Only an abdominal drain was placed into the subhepatic area. Probably, a posteriori, in addition to the abdominal drain, a T tube placement through the cystic stump, at this time, would be the safest thing to do, with the aim of draining the CBD more effectively and performing cholangiography during the postoperative period more easily in different oblique views. CT and MR findings would be hardly interpreted in the presence of material for vertebral osteosynthesis.

Clinical deterioration with persisting flow of a bilious fluid from the abdominal drain required a reoperation in a highly specialised hepatobiliary surgical Division. In front of a high index of suspicion of CBD leakage, only a cholangiography performed in different oblique views permitted the visualisation of bile leakage.

The principles of operative management in the unstable patient follow the guidelines of damage control laparotomy. These include control of hemorrhage, prevent of contamination, and avoidance of intraoperative metabolic failure. The rule is to move these patients to the intensive care unit rapidly to stabilize their physiology before subsequent definitive repair [30]. In the stable patient, the initial laparotomy affords the best opportunity for the diagnosis and definitive repair of CBD injuries. With as little as 24 hours of gross contamination, inflammatory changes develop and may not only limit surgical options but also predispose to the development of further complications [31].

The treatment options for an extrahepatic biliary leak have broadened. Until recently, such injuries usually mandated surgical repair utilizing debridement and closure with or without T-tube; patch closure using gallbladder, cystic duct, vein, serosa or jejunum; biliary enteric anastomosis using duodenum or jejunum; or ligation and drainage with plans for subsequent enteric diversion [32]. When the only relative indication for surgery is the bile leak, nonoperative management is possible [33].

**Figure 2:**
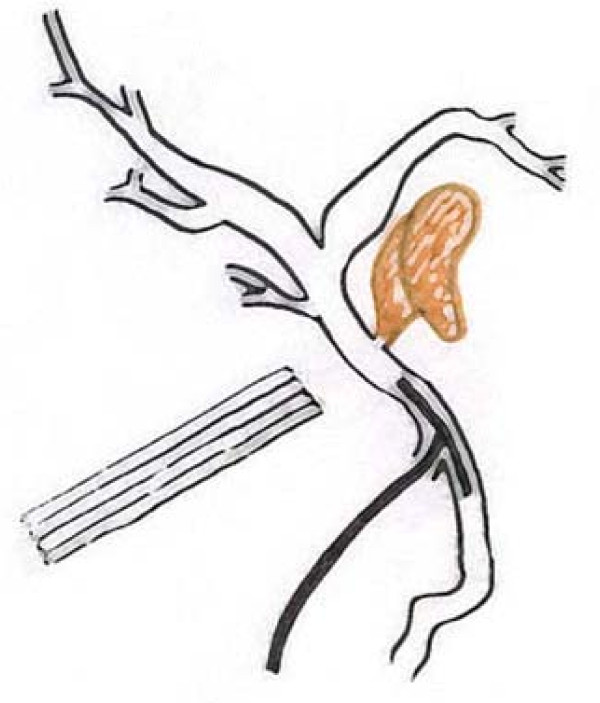
**Surgical management of the biliary leakage.** An abdominal drain is placed into the *porta hepatis * area. A T-tube is placed in the choledochus through the residual cystic duct stump. Biliary leakage, on the left posterolateral aspect of the common bile duct, 1 cm below the biliary confluence, is highlighted in yellow.

In our case, during the last intervention, because of a biliary peritonitis and inflammatory changes due to the late diagnosis, the dissection of CBD and the direct approach to the biliary leak was considered dangerous and not indicated; only the achievement of an external biliary fistula, well drained, was possible; therefore, a T-tube was placed in the choledochus through the residual cystic duct stump, and not through the biliary leakage who was at the opposite and inaccessible aspect of the common bile duct. Also an abdominal drain was placed into the subhepatic region (Figure 2). This allowed to achieve a well drained external fistula, and consequently to dry up the biliary leak one month later. Our patient returned to full activity, had normal serum hepatic enzyme levels and no sequelae from her injury.

## Conclusions

We present a case of an isolated extrahepatic bile duct rupture in blunt abdominal trauma. A literature review was conducted to detect all similar cases. Many few cases were found. Common bile duct injury is often discovered immediately during laparotomy. The diagnosis of a bile duct injury is often difficult in the multiply injured patient. The combination of suboptimal imaging modalities, the presence of confounding injuries, and the rare incidence of blunt traumatic CBD injuries contribute to the diagnostic challenge of these problems. Late recognition and inappropriate management of these injuries result in severe, often fatal consequences. The approach to the management of these patients depends primarily on the patient’s hemodynamic status. The principles of operative management in the unstable patient follow the guidelines of damage control laparotomy. The treatment options for an extrahepatic biliary leak have broadened.

## Consent

Written informed consent was obtained from the patient for publication of this Case report and any accompanying images. A copy of the written consent is available for review by the Editor-in-Chief of this journal.

## Competing interests

The authors declare that they have no competing interests.

## Authors’ contributions

BR and SC made substantial contributions to conception and design. CS and CO have been involved in drafting the manuscript or revising it critically. ZG made substantial contribution to the review. All authors read and approved the final manuscript.
